# MRI-based patient-specific human carotid atherosclerotic vessel material property variations in patients, vessel location and long-term follow up

**DOI:** 10.1371/journal.pone.0180829

**Published:** 2017-07-17

**Authors:** Qingyu Wang, Gador Canton, Jian Guo, Xiaoya Guo, Thomas S. Hatsukami, Kristen L. Billiar, Chun Yuan, Zheyang Wu, Dalin Tang

**Affiliations:** 1 School of Biological Science and Medical Engineering, Southeast University, Nanjing, China; 2 Department of Mechanical Engineering, University of Washington, Seattle, WA, United States of America; 3 Department of Mathematics, Southeast University, Nanjing, China; 4 Division of Vascular Surgery, University of Washington, Seattle, WA, United States of America; 5 Biomedical Engineering Department, Worcester Polytechnic Institute, Worcester, MA, United States of America; 6 Department of Radiology, University of Washington, Seattle, WA, United States of America; 7 Mathematical Sciences Department, Worcester Polytechnic Institute, Worcester, MA, United States of America; Massachusetts Institute of Technology, UNITED STATES

## Abstract

**Background:**

Image-based computational models are widely used to determine atherosclerotic plaque stress/strain conditions and investigate their association with plaque progression and rupture. However, patient-specific vessel material properties are in general lacking in those models, limiting the accuracy of their stress/strain measurements. A noninvasive approach of combining in vivo 3D multi-contrast and Cine magnetic resonance imaging (MRI) and computational modeling was introduced to quantify patient-specific carotid plaque material properties for potential plaque model improvements. Vessel material property variation in patients, along vessel segment, and between baseline and follow up were investigated.

**Methods:**

In vivo 3D multi-contrast and Cine MRI carotid plaque data were acquired from 8 patients with follow-up (18 months) with written informed consent obtained. 3D thin-layer models and an established iterative procedure were used to determine parameter values of the Mooney-Rivlin models for the 81slices from 16 plaque samples. Effective Young’s Modulus (YM) values were calculated for comparison and analysis.

**Results:**

Average Effective Young’s Modulus (YM) and circumferential shrinkage rate (C-Shrink) value of the 81 slices was 411kPa and 5.62%, respectively. Slice YM value varied from 70 kPa (softest) to 1284 kPa (stiffest), a 1734% difference. Average slice YM values by vessel varied from 109 kPa (softest) to 922 kPa (stiffest), a 746% difference. Location-wise, the maximum slice YM variation rate within a vessel was 311% (149 kPa vs. 613 kPa). The average slice YM variation rate for the 16 vessels was 134%. The average variation of YM values for all patients from baseline to follow up was 61.0%. The range of the variation of YM values was [-28.4%, 215%]. For plaque progression study, YM at follow-up showed negative correlation with plaque progression measured by wall thickness increase (WTI) (r = -0.7764, p = 0.0235). Wall thickness at baseline correlated with WTI negatively, with r = -0.5253 (p = 0.1813). Plaque burden at baseline correlated with YM change between baseline and follow-up, with r = 0.5939 (p = 0.1205).

**Conclusion:**

In vivo carotid vessel material properties have large variations from patient to patient, along the diseased segment within a patient, and with time. The use of patient-specific, location specific and time-specific material properties in plaque models could potentially improve the accuracy of model stress/strain calculations.

## 1. Introduction

Cardiovascular disease is a major cause of death in the world [1]. A large number of fatal clinical events, such as strokes and heart attacks are caused by vulnerable atherosclerotic plaque rupture [2–5]. It is believed that mechanical forces play a very important role in plaque progression and rupture processes [6]. With the advances of medical imaging technologies [7–8], image-based computational models have been introduced to calculate plaque stress/strain conditions and investigate their association with plaque progression and rupture [9–20]. However, the accuracy of the computational results is heavily dependent on the data and assumptions used by those models. Data needed for image-based plaque computational models include: a) plaque morphology and components; b) vessel and plaque component material properties; and c) blood flow and pressure conditions [6]. While most researchers used patient-specific plaque morphology data, their computational models lack patient-specific vessel material properties [6, 9–23]. Non-invasive techniques to obtain in vivo patient-specific vessel material properties are needed to further improve in vivo image-based plaque models [24–26].

Considerable efforts have been made by several research groups to quantify mechanical material properties of atherosclerotic vessels. Holzapfel et al. used cyclic quasistatic uniaxial tension tests in axial and circumferential directions for different components of atherosclerotic lesions from human atherosclerotic iliac arteries [27]. Anisotropic and highly nonlinear tissue components properties as well as considerable interspecimen differences were identified by the experimental data of individual samples. The circumferential direction of the fibrous cap demonstrated the lowest fracture stress (254.8 +/- 79.8 kPa at stretch 1.182 +/- 0.1) of all intimal tissues. The adventitia exhibited the highest and the nondiseased media the lowest mechanical strength on average [27]. Different layers displayed different direction-dependent mechanical behaviors, which are of vital importance to the realistic computational models and accurate stress/strain prediction [16, 27–28]. Williamson et al. reported that artery wall stresses have low sensitivities for material properties variability [29]. In a histology-based model, a +/- 50% variation in elastic modulus was found to lead to less than a 10% change in artery wall stress at the site of rupture [29]. Tang et al. investigated the effects of plaque structure and material properties on stress behaviors in human atherosclerotic plaques by using 3D FSI models, and reported that the softer materials result in higher stress values [30]. Teng et al. performed uniaxial tests using different plaque components, axial and circumferential oriented adventitia, media and intact specimens prepared from human carotid arteries [31–32]. Most of the material properties investigations in the literature used ex vivo specimens and in vitro experimental techniques. Nieuwstadt et al. performed a numerical feasibility study for carotid plaque elasticity estimation using ultrasound elastography, MRI, and inverse FEA [24]. They were able to estimate material coefficients for carotid intima and lipid-rich necrotic core (lipid) with histology validations. Smoljkić et al. proposed a non-invasive, energy-based assessment of patient-specific material properties of arterial tissue [25]. Their results showed that imposing conditions on strain energy can provide a good estimation of carotid material properties from the non-invasively measured pressure and diameter data. Czernuszewicz et al. performed some preliminary study of non-invasive in vivo characterization of human carotid plaques with acoustic radiation force impulse ultrasound. Their method was able to differentiate soft tissues from stiffer tissues with histological validations [26]. In vivo vessels material properties and patient-specific material properties are still scarce in the current literature.

For plaque models based on in vivo image data, shrinkage rate (axial shrinkage and circumferential shrinkage) should be determined so that the in vivo vessel geometry could be shrunk to its “no-load” shape from which loaded stress/strain conditions could be obtained. Tang et al. and Yang et al. introduced a shrink-stretch process in their 3D fluid-structure interaction (FSI) models with in vivo plaque data [16, 33–35]. Their shrink-stretch process include: a) axial and circumferential shrink the vessel to get the numerical starting geometry; and b) axial stretch and circumferential pressurization were applied to recover the vessel in vivo shape. The axial shrinkage was 9% so that the in vivo vessel length could be recovered when a 10% axial stretch were applied. The Circumferential shrinkages of vessel lumen and outer wall were determined so that: a) the vessel volume was conserved; b) vessel shape after pressurization and 10% axial stretch recovered the original in vivo shape. Speelman et al. and Gee et al. also showed the importance and necessity of the pre-shrink process with their computed tomography (CT)-based simulations for abdominal aortic aneurysm [36–37]. Huang et al. reported a non-uniform shrink-stretch process which had better match with the in vivo vessel geometries [38]. Liu et al. introduced a non-invasive approach to quantify patient-specific vessel material properties and plaque circumferential shrinkage rate between vessel in vivo and “no-load” geometries [39]. Their material properties and circumferential shrinkage rate were calculated by 2D plaque models. Their results showed that effective Young’s Modulus (YM) from the 12 human carotid arteries varied from 137 kPa to 1435 kPa and vessel circumferential shrinkage to “no-load” condition varied from 6% to 32%. Overall, quantified patient-specific shrinkage rate using in vivo data are rare in the current literature.

In this paper, a non-invasive approach [39] of combining 3D multi-contrast MRI, in vivo Cine MRI and computational 3D thin-layer model [40] was used to quantify patient-specific carotid plaque material properties and circumferential shrinkage rates. The variation of the material properties among different subjects, within each diseased artery, and with time was investigated.

## 2. Methods

### 2.1 In vivo serial MRI data acquisition and segmentation

**Fig 1:**
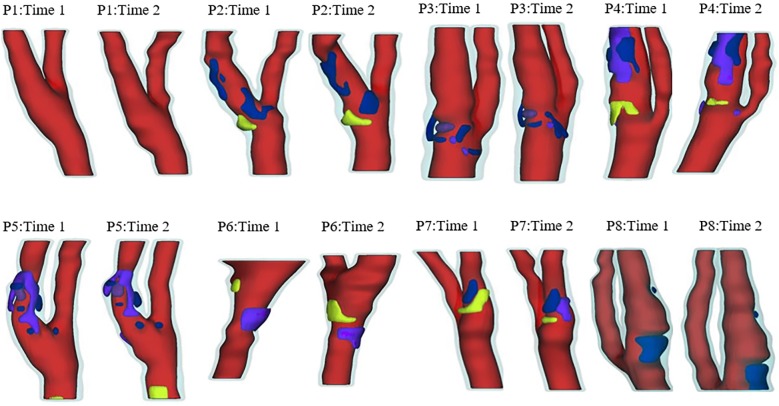
3D plots of re-constructions 3D geometries of the 16 plaques (8 time pairs). Red: Lumen; Light blue: Vessel; Yellow: Lipid-rich core; Blue: Calcification; Dark Blue: Loose Matrix.

**Fig 2:**
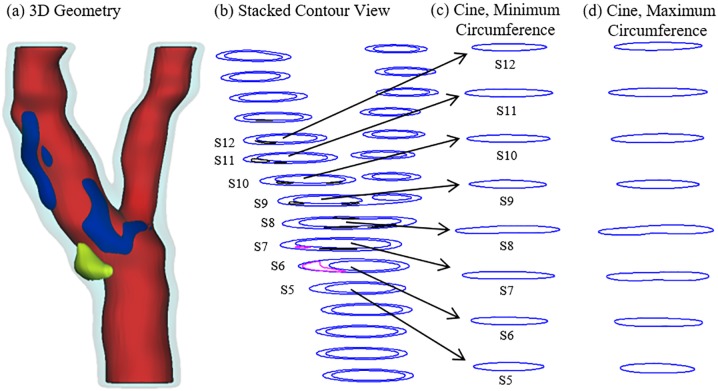
A human carotid plaque sample with matching Cine slices. (a) 3D plaque geometry reconstructed from MRI; (b) Stacked contours; (c) Matching Cine contours with minimum circumference; (d) Matching Cine contours with maximum circumference.

Serial MRI data of carotid atherosclerotic plaques from 8 patients (5 male, 3 female; age: 62–83, mean = 71; see Table 1 for details) were acquired at the University of Washington (UW), Seattle by the Vascular Imaging Laboratory (VIL) using protocols approved by the UW Institutional Review Board and with written informed consent obtained. For each patient, MRI slices at baseline (Time 1, T1) and follow-up (Time 2, T2, Scan time intervals were about 18 months) were matched up using vessel bifurcation, stenosis features and with careful review by the MRI group. For simplicity, the vessel segment assembled using the selected slices is called the plaque. Cuff systolic and diastolic arm pressure was recorded for modeling use. In vivo Cine and three-dimensional (3D) multi-contrast MR images of the carotid arteries were acquired using a 3.0T whole-body scanner (Philips Achieva, R2.6.1, Best, The Netherlands) and a dedicated 8-channel, phased array carotid coil. The carotid bifurcation was located on two-dimensional (2D) TOF (Time of Flight) and oblique black blood MR images. A 3.5cm region centered on the carotid bifurcation was imaged by high-resolution axial bright and black blood imaging. Detailed data acquisition and segmentation procedures were published before and are omitted here [10, 39]. For each patient, locations with Cine sequence and nearly-circular slices from 3D MRI were selected for calculating the material parameter values in the modified Mooney-Rivlin model used in our previous publications. Fig 1 gives plots of 16 re-constructed carotid plaques based on in vivo MRI data from 8 patients, each with 2 time points. Fig 2 gives a sample plaque with 3D MRI slices and matching Cine lumen contours at multiple locations corresponding to minimum and maximum pressure conditions. The Cine and 3D MRI sequences were designed with equal slice distance and matched using longitudinal position from the bifurcation.

**Table 1:** Patient information and pressure data at baseline and follow-up when available (Scan time intervals were about 18 months; L: left carotid artery; R: right carotid artery).

Patients	Gender	Age	Weight (lbs)	Height	Pressure(mmHg)	Index Side
1	Female	66	106	4'11"	T1 and T2 (120,80)	L
2	Male	76	170	5'10"	T1(120,70); T2(141,72)	L
3	Male	62	185	6'0"	T1(130,70); T2(143,80)	L
4	Female	63	161	5'6''	T1 and T2 (146,81)	L
5	Male	63	140	6'2"	T1 and T2 (100,60)	L
6	Male	83	180	5'8"	T1 and T2 (143,73)	R
7	Female	70	140	5'7''	T1 and T2 (143,90)	L
8	Male	81	180	5'6"	T1 and T2 (143,65)	R

### 2.2 The 3D thin-layer model to determine material parameter values

**Fig 3:**
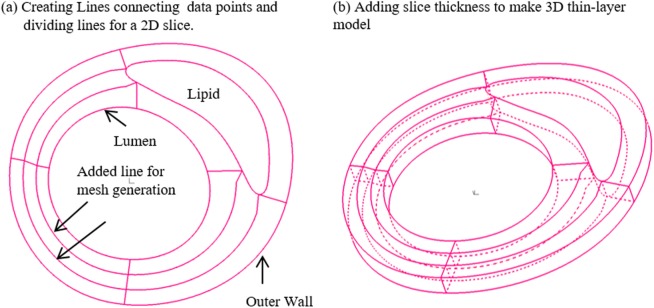
3D thin-layer model construction. (a) Creating Lines connecting data points and dividing lines for a 2D slice; (b) Adding slice thickness to make 3D thin-layer model.

A 3D thin-layer modeling approach introduced by Huang et al. [40] was used to determine material parameter values in our selected material model. For every slice that Cine data was available, a thin slice thickness was added to make a 3D thin-layer model (see Fig 3). Then our established 3D model construction and mesh generation procedures with axial and circumferential shrink were applied in the material value determination process [40]. The carotid artery was assumed to be hyperelastic, isotropic, incompressible and homogeneous. The nonlinear modified Mooney-Rivlin (M-R) model was selected to describe the material properties of the vessel wall [41–42]. The strain energy function was given by:

$$\text{W}={\text{c}}_{1}\left({\text{I}}_{1}-3\right)+{\text{c}}_{2}\left({\text{I}}_{2}-3\right)+{\text{D}}_{1}\left[\text{exp}\left({\text{D}}_{2}\left({\text{I}}_{1}-3\right)\right)-1\right],$$ (1)

$$I_{1}=\sum C_{ii},\;I_{2}=\frac{1}{2}[I_{1}^{2}-C_{ij}C_{ij}],$$ (2)

where C = [C_ij_] = X^T^X is the right Cauchy-Green deformation tensor; I_1_ and I_2_ are the invariants of C; $X=[X_{ij}]=\left[\frac{\partial x_{i}}{\partial a_{j}}\right]$ is the deformation gradient; c_1_, c_2_, D_1_ and D_2_ form the material parameter set. The modified Mooney-Rivlin model was selected because it was able to fit carotid artery vessel properties measured by uniaxial and biaxial mechanical testing data and good agreement was obtained [9, 43]. With our limited pressure data (only two pressure data points: maximum and minimum pressures), material parameter values could not be uniquely determined. According to our previous experiences [10, 16], we set c_2_ = 0 and D_2_ = 2. The c_1_/D_1_ value was adjusted. For lipid cores, we used parameter values in our previous publications: c_1_ = 0.5 kPa, D_1_ = 0.5 kPa, D_2_ = 0.5 [10].

**Fig 4:**
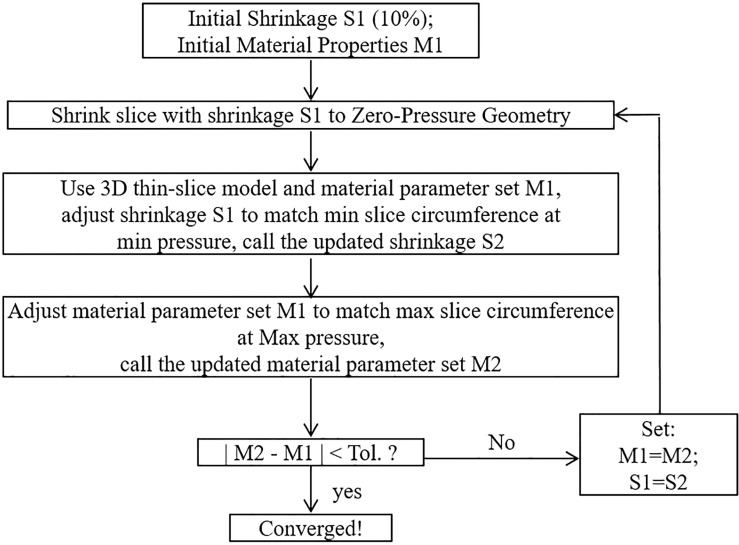
The flow chart for the iterative procedure to determine material parameters and vessel circumferential shrinkage rate.

For each 3D thin-layer model, a 10% axial shrinkage rate was applied. Then an iterative procedure [39] was followed to adjust the parameter values in the modified M-R model and the circumferential shrinkage rate to match both maximum and minimum Cine lumen circumferences corresponding to systolic and diastolic pressures, respectively (see Fig 4 for details). Material parameters values c_1_ (36.6 kPa) and D_1_ (14.4kPa) from our previous paper [10] were used as initial estimates for each slice. The initial lumen circumferential shrinkage rate S_1_ was set to be 10%. The details of the iteration procedure are given in Fig 4. The 3D thin-layer model for each iteration was solved by ADINA (ADINA R & D, Watertown, MA).

### 2.3 The 2D models for comparison purpose

To compare our 3D thin-layer model approach with 2D model approach, parameter values for Modified Mooney-Rivlin (MR) models and circumferential shrinkage values for the 81 slices were also calculated using 2D modeling method [29] with the iterative procedure given in Fig 4. The main difference between 2D model and 3D thin-layer model is that 2D model does not have axial stretch while 3D thin-layer model does which makes the 3D thin-layer model closer to full 3D models. Their differences are reported in Section 3.5.

### 2.4 Using an effective Young’s modulus as a stiffness indicator for material properties

The stress-stretch relationship for the Mooney-Rivlin model is given by:

$$\sigma =\lambda \frac{dW}{d\lambda }=2\lambda (\lambda -\lambda^{-2})\left(c_{1}+D_{1}D_{2}e^{D_{2}(\lambda^{2}+\frac{2}{\lambda }-3)}\right),$$ (3)

where σ is Cauchy stress, and λ is stretch ratio. In order to facilitate comparison, it is easier to use a single parameter to compare vessel stiffness from different patients or slices. The effective Young’s modulus (YM) E for the stretch ratio interval [1.0, 1.3] is defined as:

σ=E(λ−1), (4)

The least-squares technique was used to calculate the YM values that best fit the M-R model.

### 2.5 Statistical method

For all correlation study related variables in Section 3.4, Shapiro-Wilk normality test showed no evidence of non-normal distribution. Standard student t-test was performed for possible correlations between plaque progression and material stiffness, plaque burden and wall thickness using our patient follow-up data. We also applied the Kolmogorov-Smirnov test to check the normality. A p-value 0.81 was obtained, still indicating no evidence of non-normality. In case the normality assumption was a concern, we calculated the correlation p-values under the non-parametric Spearman rank test (see 3.4), which does not require the normality assumption. The results were consistent with those by the parametric t-test.

## 3. Results

### 3.1 Overview of material parameter values, effective YM and circumferential shrinkage rates

**Fig 5:**
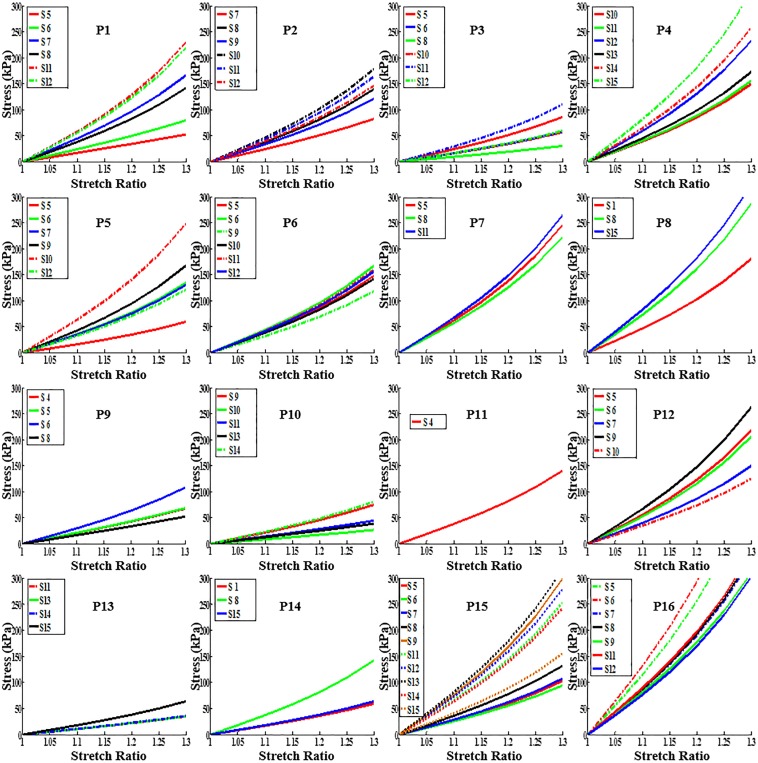
Stress-stretch curves from Mooney-Rivlin Models using parameter values determined from Cine MRI for the 16 plaque samples studied.

Fig 5 presents stress-stretch ratio plots of the 81 slices from the 16 plaque samples. For each plaque, choosing the stiffest (with maximum YM) and softest (with minimum YM) slices as representatives, the M-R model parameter values (C_1_ and D_1_), maximum and minimum Cine lumen circumferences (Cir_-Max_ and Cir_-Min_), relative lumen circumferences variation rates (δ_-Cir_), maximum and minimum lumen pressure (P), effective Young’s modulus (YM) and circumferential shrinkage rate (C-Shrink) were provided by Table 2. The relative lumen circumferences variation rate (δ_-Cir_) is defined as:

$$\delta_{\text{-}Cir}=\frac{{Cir}_{\text{-}Max}-{Cir}_{\text{-}Min}}{{Cir}_{\text{-}Max}},$$ (5)

**Table 2:** Maximum and minimum circumferences, pressure, material parameter and circumferential shrinkage values of the softest and stiffest slices from 16 plaque samples.

Plaque	Slice	Cir_-Max_	Cir_-Min_	δ_-Cir_	P	C1	D1	YM	C-Shrink
		(cm)	(cm)	(%)	(mmHg)	(kPa)	(kPa)	(kPa)	(%)
P1	Softest	2.374	2.003	15.6	(120,80)	27.1	0.47	139	15.3
	Stiffest	2.025	1.944	4.01		37.0	27.9	564	1.59
P2	Softest	3.086	2.744	11.1	(120,80)	31.0	4.39	212	13.8
	Stiffest	1.970	1.877	4.76		36.5	19.2	443	2.87
P3	Softest	2.936	2.498	14.9	(120,70)	13.0	1.10	78.8	19.7
	Stiffest	2.420	2.197	9.23		25.0	11.1	275	6.67
P4	Softest	2.352	2.141	8.97	(141,72)	30.0	16.3	370	3.19
	Stiffest	2.095	1.988	5.15		50.0	40.2	797	-1.11
P5	Softest	2.460	2.160	12.2	(130,70)	15.0	5.52	149	12.6
	Stiffest	2.017	1.906	5.50		45.0	28.6	613	0.09
P6	Softest	2.275	2.038	10.4	(143,80)	30.0	10.9	296	7.29
	Stiffest	2.167	2.030	6.34		36.0	17.4	416	1.70
P7	Softest	2.509	2.355	6.16	(146,81)	40.0	25.6	548	1.31
	Stiffest	3.172	2.931	7.59		45.0	31.4	651	3.75
P8	Softest	2.553	2.321	9.12	(146,81)	35.0	20.1	448	4.87
	Stiffest	1.485	1.430	3.68		60.0	36.6	797	-1.55
P9	Softest	1.659	1.441	13.2	(100,60)	25.0	1.10	138	9.87
	Stiffest	2.850	2.638	7.46		30.0	9.11	272	3.83
P10	Softest	1.080	0.922	14.6	(100,60)	13.0	0.45	69.9	13.8
	Stiffest	1.445	1.320	8.65		25.0	6.03	205	5.78
P11	only one	2.136	1.906	10.8	(143,73)	35.0	13.1	352	4.56
P12	Softest	3.863	3.327	13.9	(143,73)	35.0	10.5	316	8.44
	Stiffest	2.955	2.746	7.09		46.0	30.7	647	0.84
P13	Softest	1.524	1.285	15.7	(143,90)	15.0	1.22	90.3	21.5
	Stiffest	1.468	1.287	12.3		20.0	4.66	162	15.7
P14	Softest	2.396	2.091	12.8	(143,90)	20.0	3.86	151	21.1
	Stiffest	1.994	1.856	6.90		30.0	15.0	353	5.77
P15	Softest	2.370	2.085	12.0	(143,65)	25.0	8.32	237	3.83
	Stiffest	1.618	1.545	4.54		60.0	35.5	782	-2.34
P16	Softest	1.775	1.662	6.34	(143,65)	50.0	36.3	744	-0.93
	Stiffest	2.546	2.443	4.05		85.0	63.2	1284	-2.74
All	Ave	2.192	1.997	8.91	(133,74)	35.5	17.3	411	5.62

Note: c_2_ = 0 and D_2_ = 2 were set for all cases. Ave: average from all 81 slices. Due to axial shrink applied to the 3D thin-layer model, some C-Shrink values in 3D thin-layer model were negative.

The averaged YM value of the 81 slices was 411kPa, which was consistent with the current literature [10–11, 28, 32]. The averaged C-Shrink and δ_-Cir_ value of the 81 slices was 5.62%, 8.91%, respectively. Strong correlations were observed: a) negative correlation between δ_-Cir_ values and YM values (r = -0.8083, p<0.0001); b) positive correlation between δ_-Cir_ values and C-Shrink values (r = 0.8537, p<0.0001; c) negative correlation between YM and C-Shrink values (r = -0.8005, p<0.0001).

### 3.2. Plaque material properties have large patient-to-patient variations

**Fig 6:**
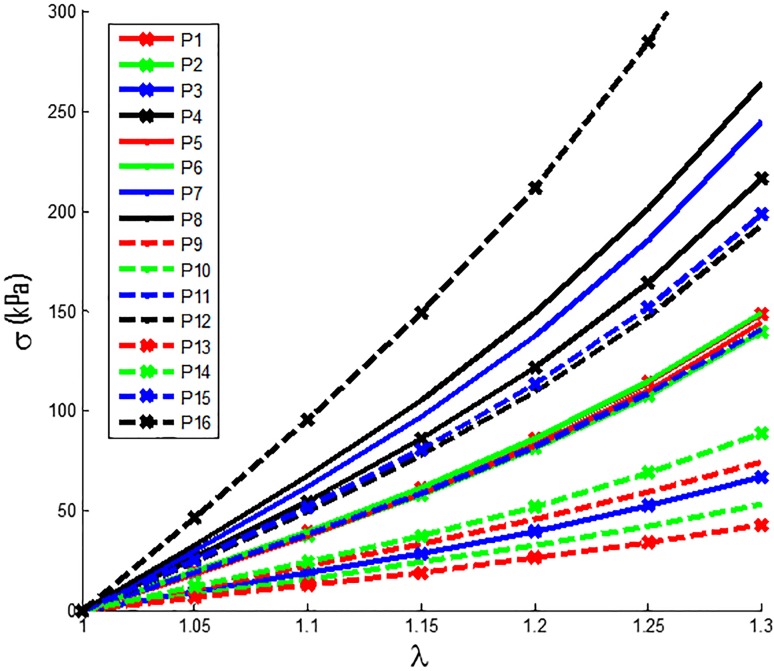
Stress-Stretch curves of Mooney-Rivlin Models for the 16 plaque samples. Each curve used average parameter values for all available slices for the given plaque sample.

Stress-Stretch Ratio curves from Mooney-Rivlin models for the 16 plaque samples using average parameter values of their slices are presented in Fig 6. The average YM values and circumferential shrinkage (C-Shrink) values from 16 plaque samples were given in Table 3. The average YM values for the stiffest plaque sample (P16) was 922 kPa, 746% higher than that for the softest plaque (P13, YM = 109 kPa). This showed that plaque material properties have large variations from patient to patient and patient-specific material properties should be used in plaque models. It should be noted that pressure difference had a big effect on the material properties of vessels. Average C-shrink value from the 16 samples was 6.29%. The softest sample had 21.7% C-shrink, while the stiffest sample had a negative C-Shrink value (-1.78%). Negative C-Shrink means to obtain the zero-load geometry of the 3D thin-layer model, the in vivo slice lumen needed to expand slightly so that it could regain the in vivo circumference when 10% axial stretch and pressure were applied. Axial stretch makes the vessel to shrink in radial direction.

**Table 3:** Average material parameter values and circumferential shrinkage for 16 human carotid plaque samples based on Cine MRI data. Due to axial shrink applied to the 3D thin-layer model, some C-Shrink values in 3D thin-layer model were negative.

Plaque	Cir_-Max_	Cir_-Min_	δ_-Cir_	P	C_1_	D_1_	YM	C-Shrink
	(cm)	(cm)	(%)	(mmHg)	(kPa)	(kPa)	(kPa)	(%)
P1	2.564	2.350	8.35	(120,80)	34.6	14.6	370	7.40
P2	2.328	2.163	7.11	(120,80)	34.8	12.9	348	6.26
P3	2.415	2.119	12.3	(120,70)	19.5	5.31	169	12.3
P4	2.208	2.050	7.15	(141,72)	37.5	25.3	531	1.05
P5	2.089	1.903	8.90	(130,70)	30.0	15.3	357	4.51
P6	2.130	1.950	8.44	(143,80)	34.3	14.7	371	4.11
P7	2.952	2.747	6.93	(146,81)	41.7	28.9	601	2.45
P8	2.073	1.942	6.33	(146,81)	48.3	30.1	650	1.07
P9	2.341	2.094	10.6	(100,60)	28.8	3.66	191	7.48
P10	1.301	1.150	11.6	(100,60)	20.6	2.61	137	9.88
P11	2.136	1.906	10.8	(143,73)	35.0	13.1	352	4.56
P12	2.658	2.389	10.1	(143,73)	38.2	21.1	476	3.96
P13	1.537	1.306	15.0	(143,90)	16.3	2.16	109	21.7
P14	2.018	1.801	10.8	(143,91)	23.3	7.84	222	14.7
P15	2.127	1.933	9.11	(143,65)	41.5	21.0	492	0.92
P16	2.191	2.075	5.28	(143,65)	66.4	43.4	922	-1.78
Ave by Patient	2.192	1.992	9.3	(133,74)	34.4	16.4	394	6.29
Ave by Slice	2.192	1.997	8.91	(133,74)	35.5	17.3	411	5.62

### 3.3. Plaque material properties change with location: slice-specific material properties

Stress-Stretch plots for all the slices from 16 plaque samples were already presented by Fig 5. It is clear that slices at different locations had different material properties. Table 4 lists the effective Young’s modulus (YM) values for all slices from the 16 plaque samples. Our results showed that the arterial segment downstream of the bifurcation (average YM value is 536 kPa) is stiffer than the common carotid artery segment (the average YM value is 321 kPa). For comparison purpose, the variation of YM (δ_-YM_) for all slices in a plaque is defined as:

$$\delta_{\text{-}YM}=({YM}_{Max}-{YM}_{Min})/{YM}_{Min},$$ (6)

**Table 4:** The YM values for all slices from 16 plaque samples based on Cine MRI data.

Plaque	YM (kPa)																δ_-YM_
	S-5	S-4	S-3	S-2	S-1	Bi-	S1	S2	S3	S4	S5	S6	S7	S8	S9	S10	(%)
P1	139	207	416	355	-	-	564	538	-	-	-	-	-	-	-	-	306
P2	-	-	-	212	349	307	443	408	367	-	-	-	-	-	-	-	109
P3	-	-	218	146	79		144	275	150	-	-	-	-	-	-	-	248
P4	-	-	-	-	-	-	370	385	572	429	632	797	-	-	-	-	115
P5	-	-	-	-	149	336	325	413	613	304	-	-	-	-	-	-	311
P6	-	-	369	416	-	-	296	356	397	389	-	-	-	-	-	-	41.0
P7	-	-	-	604	-	-	548	-	-	651	-	-	-	-	-	-	19.0
P8	448	-	-	-	-	-	706	-	-	-	-	797	-	-	-	-	78.0
P9	-	-	176	178	272	-	138	-	-	-	-	-	-	-	-	-	97.0
P10	-	-	-	-	-	-	191	70	116	102	205	-	-	-	-	-	193
P11	352	-	-	-	-	-	-	-	-	-	-	-	-	-	-	-	0.0
P12		537	508	374	647	316	-	-	-	-	-	-	-	-	-	-	105
P13	-	-	-	-	-	-	90 .0	91.0	94 .0	162	-	-	-	-	-	-	80.0
P14	151	-	-	-	-	353	-	-	-	-	162	-	-	-	-	-	134
P15	-	-	-	-	-	257	237	269	332	735	-	625	692	782	602	386	230
P16	-	-	-	-	-	-	-	-	1126	1284	828	841	769	-	861	744	73.0
Ave.	273	372	337	326	299	314	338	312	419	507	457	765	731	782	732	565	134

Note: Bi- is the bifurcation. c_2_ = 0 and D_2_ = 2 for all cases.

δ_-YM_ is the YM variation rate of the stiffest slice over the softest slice. The biggest δ_-YM_ values for the slices from one vessel (P5) was 311%. The smallest δ_-YM_ value for the slices from one vessel (P7) was 19.0%. The YM values variation of 13 plaque samples from 16 plaque samples were more than 50.0%. It showed that material properties change considerably along the artery segments and tend to become stiffer at downstream side. Location-specific material properties should be used when available in image-based plaque models for improved accuracy of the plaque models.

### 3.4 Plaque material properties change over time

We investigated whether vessel stiffness changed over time, and how material stiffness changes were associated with plaque progression and other risk factors. The effective Young’s modulus (YM), wall thickness (WT) [44], plaque burden (PB) and circumferential shrinkage (C-shrink) values at baseline (T1) and follow-up (T2) for the 8 patients are given in Table 5. The variation of YM over time (δ_YM-Time_), the difference of PB (δ_PB_) and the WT increase (WTI) are given by:

$$\delta_{YM\text{-}Time}=({YM}_{T2}-{YM}_{T1})/{YM}_{T1},$$ (7)

$$\delta_{PB}={PB}_{T2}-{PB}_{T1},$$ (8)

$$WTI={WT}_{T2}-{WT}_{T1},$$ (9)

where YM_T1_, PB_T1_ and WT_T1_ are the YM, PB and WT values at T1, and YM_T2_, PB_T2_ and WT_T2_ are the YM, PB and WT values at T2, respectively. The average δ_YM-Time_, δ_PB_ and WTI values in Table 5 were calculated using absolute values since otherwise positive and negative changes could cancel against each other give misleading lower averaged changes. The average variation of YM values for all patients was 61.0%. The range of variation of YM values was [-28.4%, 215%]. 3 patients had increased PB while 5 showed PB decrease. The average PB change for the 8 patients was 6.11%. 3 patients had positive WTI, while 5 had negative WTI. The range of WTI was [-0.225, 0.216] (mm). YM at T1 could have positive correlation with YM at T2 but the statistical significance is not strong (Pearson's product-moment correlation coefficient r = 0.7030, p = 0.0518 under t-test, and p = 0.1323 under Spearman’s rank test). YM at T2 showed strong negative correlation with WTI, with r = - 0.7764 (p = 0.0235 under t-test and p = 0.0154 under Spearman’s rank test). WT at T1 seems to be negatively correlated with WTI, but cannot statistically confirmed yet (r = -0.5253, p = 0.1813 under t-test and p = 0.1511 under Spearman’s rank test). Also, the seemly positive correlation between PB at T1 and δ_YM-Time_ cannot be statistically confirmed yet (r = 0.5939, p = 0.1205 under t-test and p = 0.0962 under Spearman’s rank test). No evidences show that the data distributions of these variables departure from Gaussian because the Shapiro-Wilk normality test p-values are all significantly larger than 0.05. Larger patient size will potentially lead to better confirmation of the variable correlations.

**Table 5:** The time-specific material YM values for carotid atherosclerotic plaques for 8 patients based on Cine MRI data by using thin-layer model.

	Time 1				Time 2				Variation		
Pts	δ_-Cir_	PB	WT	YM	δ_-Cir_	PB	WT	YM	δ_-PB_	WTI	δ_YM-Time_
	(%)	(%)	(mm)	(kPa)	(%)	(%)	(mm)	(kPa)	(%)	(mm)	(%)
1	8.35	32.0	0.911	370	7.11	34.2	0.883	348	2.11	-0.028	-5.95
2	12.3	49.4	1.337	169	7.15	46.5	1.145	531	-2.90	-0.192	215
3	8.90	47.3	1.126	357	8.44	42.1	1.059	371	-5.2	-0.067	3.87
4	6.93	43.9	1.364	601	6.33	49.8	1.327	650	5.90	-0.037	8.17
5	10.6	39.4	1.077	191	11.6	64.1	1.293	137	24.7	0.216	-28.4
6	10.8	39.0	0.872	352	10.1	38.0	1.016	476	-0.98	0.144	35.5
7	15.0	49.7	0.980	109	10.8	44.3	1.159	222	-5.34	0.179	103
8	9.11	40.2	1.098	492	5.28	38.4	0.873	922	-1.76	-0.225	87.5
*Ave.	10.2	42.6	1.096	330	8.35	44.7	1.094	457	6.11	0.136	61.0

Pts is patients. PB is plaque burden. WT is wall thickness. WTI is wall thickness increase.

*: The average δYM-Time, δPB and WTI values were calculated using absolute values. c_2_ = 0 and D_2_ = 2 for all cases. Due to axial shrink applied to the 3D thin-layer model, some C-Shrink values in 3D thin-layer model were negative.

Data normality was checked by the Shapiro-Wilk test. For the YM data over time in Table 5, it has a Shapiro-Wilk p-value 0.17, indicating no evidence of non-normality. We also applied the Kolmogorov-Smirnov test to check the normality. We got p-value 0.81, still indicating no evidence of non-normality. When the Kolmogorov test is applied, data have to be re-scaled based on its mean and standard deviation. If the YM data were not re-scaled, the Kolmogorov test would give a small p-value. Since our data size is small and the normality assumption could be a concern, we also reported the correlation p-values under the non-parametric Spearman rank test, which does not require the normality assumption. The results are consistent with those by the parametric t-test.

### 3.5 The differences between 2D model and 3D thin-layer model

To compare our 3D thin-layer model approach with 2D model approach, parameter values for Modified Mooney-Rivlin (MR) models and circumferential shrinkage values for the 16 plaque samples from 8 patients were also calculated using 2D modeling method [29] with the iterative procedure given in Fig 4. The average effective YM and C-Shrink values for the 16 plaque samples calculated by the 2D model and 3D thin-layer model are given in Table 6. The variation of YM (δ_-YM-Model_) and the difference of C-Shrink (δ_C-Shrink_) between the 2 models are given by:

$$\delta_{\text{-}YM\text{-}Model}=({YM}_{3D}-{YM}_{2D})/{YM}_{2D},$$ (10)

$$\delta_{\text{-}C\text{-}Shrink}={C\text{-}Shrink}_{3D}-{C\text{-}Shrink}_{2D},$$ (11)

where YM_2D_ and C-Shrink_2D_ are the YM and circumferential shrinkage values, respectively, calculated by the 2D model, and YM_3D_ and C-Shrink_3D_ are the YM and circumferential shrinkage values, respectively, calculated by the 3D thin-layer model.

**Table 6:** The average effective Young’s modulus (YM) values and circumferential shrinkage values for 16 carotid plaque samples based on Cine MRI data by using 2D and 3D thin-layer model.

Plaque	2D model		3D thin-layer model		Variation	
	YM	C-Shrink	YM	C-Shrink	δ_˗YM˗Model_	δ_˗C˗Shrink_
	(kPa)	(%)	(kPa)	(%)	(%)	(%)
P1	342	12.3	370	7.40	8.38	-4.86
P2	320	11.2	348	6.26	8.44	-4.91
P3	156	16.8	169	12.3	7.98	-4.56
P4	493	6.29	531	1.05	7.99	-5.24
P5	328	9.53	357	4.51	9.04	-5.02
P6	341	9.15	371	4.11	8.74	-5.04
P7	561	7.54	601	2.45	7.11	-5.09
P8	604	6.31	650	1.07	7.91	-5.24
P9	177	12.1	191	7.48	7.85	-4.62
P10	126	14.4	137	9.88	8.90	-4.48
P11	323	9.62	352	4.56	8.85	-5.05
P12	441	9.12	476	3.96	8.00	-5.16
P13	104	25.0	109	21.7	5.19	-3.29
P14	208	19.0	222	14.7	6.85	-4.24
P15	453	6.20	492	0.92	8.67	-5.29
P16	856	3.67	922	-1.78	7.78	-5.44
Ave by Patient	365	11.1	394	6.29	7.98	-4.85
Ave (81 Slices)	380	10.5	411	5.62	8.16	-4.88
Min	104	3.67	109	-1.78	5.19	-5.44
Max	856	25.0	922	21.7	9.04	-3.29

Note: c_2_ = 0 and D_2_ = 2 for all cases. Due to axial shrink applied to the 3D thin-layer model, some C-Shrink values in 3D thin-layer model were negative. Both averages by patients and by slices are given.

YM values from the 3D thin-layer models were 7.98% higher than that from the 2D models. The range of δ_-YM-Model_ values was [5.19%, 9.04%]. C-Shrink values from the 3D thin-layer models were 4.85% lower than that from the 2D models. The range of δ_-C-Shrink_ values was [-5.44%, -3.29%]. These differences showed that using 3D thin-layer model to replace 2D model would lead to improved accuracy of material parameter value estimations.

## 4. Discussions

### 4.1. Significance of in vivo patient-specific and location-specific vessel material properties

Most of the research on determining arterial wall material properties has been performed using ex vivo specimens and in vitro experimental techniques. In vivo estimation of patient-specific material properties is scarce, which is a serious limitation for patient-specific plaque models. A noninvasive approach of combining in vivo Cine and 3D MRI and simple 3D thin-layer modeling was introduced to quantify patient-specific and location-specific vessel material properties and improve model prediction accuracies. Our results from 16 plaques and 81 slices showed that slice YM values could vary from 70 kPa to 1284 kPa, 16 times of the lowest YM value. YM values from slices of the same vessel could vary by 311% (Table 4). Future studies should render 3D plaque models using patient-specific and location-specific material properties to quantify their impact on stress/strain calculations.

### 4.2. Significance of the 3D thin-layer model

Full 3D computational models are essential for plaque stress and strain calculations. They should also be used in the iteration process to determine the material parameter values that fit the variation in luminal circumference with the cardiac cycle, as provided by imaging techniques such as MR Cine data. However, full 3D model construction is time consuming. The material parameter adjusting process takes 20–50 iterations. The labor cost is unbearable if full 3D models were used. Previously, 2D models were used as an approximation [39]. We proposed a 3D thin-layer modeling method as an approximation with much less computational cost than full 3D models. 3D axial stretch was applied to the 3D thin-layer models, mimicking full 3D model procedures. The 3D thin-layer model was closer to the real in vivo conditions than used 2D models. This 3D thin-layer approach needs less computational cost than a full 3D model to achieve convergence for both circumferential shrinkage rate and material parameter value. Compared to 2D model results, the YM values from the 3D thin-layer models were 7.98% higher. The difference showed that using 3D thin-layer model to replace 2D model would lead to improved accuracy of material parameter value estimations.

### 4.3. Vessel material properties change over time and plaque component material properties

Plaque material properties changed over time with a 61.0% average variation of YM among all patients. It is clearly of great importance to be able to quantify patient-specific, location-specific and time-specific vessel material parameter values under in vivo conditions. However, there are some limitations as the available Cine MR data did not provide plaque component size change information needed to calculate plaque component material parameter data. In this study, lipid core parameter values were extracted from previous publications [10]. New imaging protocols or in vitro experimental techniques could be used to obtain patient-specific plaque component properties.

### 4.4. Impact of hypertension

It is well-accepted that hypertension is a common thread in most cardiovascular diseases. Table 2 shows that the six patients who had higher than 140 mmHg systolic pressure had higher YM values. The average YM value for the six patients was 612 kPa, whereas the average YM value for the remaining patients was 263 kPa. Since we do not have direct pressure measurements at present time, we plan to investigate the impact of hypertension when better pressure data becomes available. And the number of patient is the important limitation for the correlation prediction between hypertension and vessel material.

### 4.5 Model limitations

Cine MRI was used to determine vessel material parameter values, matching in vivo plaque geometries under both systolic and diastolic pressure conditions. Cine MRI is widely accepted to acquire time-dependent vessel motion and deformation. Multi-layer structure and anisotropic material properties of arteries were not considered since MRI does not provide layer information. Cine data provided only circumference variations under cardiac pressure. Another limitation was that location-specific pressure measurement was not available. Currently, arm cuff pressure values are used in most image-based studies. Noninvasive acquisition of intraplaque pressure data remains a challenge.
